# Metasensor: A Proposal for Sensor Evolution in Robotics

**DOI:** 10.3390/s25030725

**Published:** 2025-01-25

**Authors:** Michele Braccini

**Affiliations:** Department of Computer Science and Engineering, University of Bologna, 47521 Cesena, Italy; m.braccini@unibo.it

**Keywords:** metasensor, sensor evolution, robotics, control by interpretation, automatic design of sensors, adaptive robots, cybernetics, biosemiotics

## Abstract

Sensors play a fundamental role in achieving the complex behaviors typically found in biological organisms. However, their potential role in the design of artificial agents is often overlooked. This often results in the design of robots that are poorly adapted to the environment, compared to their biological counterparts. This paper proposes a formalization of a novel architectural component, called a metasensor, which enables a process of sensor evolution reminiscent of what occurs in living organisms. The metasensor layer searches for the optimal interpretation of its input signals and then feeds them to the robotic agent to accomplish the assigned task. Also, the metasensor enables a robot to adapt to new tasks and dynamic, unknown environments without requiring the redesign of its hardware and software. To validate this concept, a proof of concept is presented where the metasensor changes the robot’s behavior from a light avoidance task to an area avoidance task. This is achieved through two different implementations: one hand-coded and the other based on a neural network substrate, in which the network weights are evolved using an evolutionary algorithm. The results demonstrate the potential of the metasensor to modify the behavior of a robot through sensor evolution. These promising results pave the way for novel applications of the metasensor in real-world robotic scenarios, including those requiring online adaptation.

## 1. Introduction

Since its introduction, cybernetics has been concerned with investigating the mechanisms that enable the control and adaptation of biological and artificial organisms. On the other hand, biosemiotics considers symbols and signs as essential elements in the economies of living beings. Their use allows them to communicate, adapt, and evolve within their biological and ecological environments.

The topic of sensory system evolution  naturally fits between the folds of the space traced by both the aforementioned disciplines. (By “evolution” we mean any procedure, either offline or online, that may allow sensors to be modified so as to make the robot better fitted to the environment in which it operates: so, in the following—even when not explicitly stated—it also includes processes of development, adaptation and learning that may occur during the robot’s lifetime). Indeed, sensors are part of control in that they are part of—along with controllers, actuators and environments—the causal closed-loop that composes the feedback mechanism required to achieve the desired behavior of the robot (cybernetic dimension). At the same time, being the interface between the external world and the internal system, they process—providing a first implicit interpretation—the signs coming from the external world (semiotic dimension). In a truly open-ended scenario [1,2,3,4], signs and their internal interpretations evolve as a consequence of the evolution of the nature and quality of the sensors themselves, changing the behavior of the robotic agent accordingly, and vice versa.

So, although sensory apparatus plays a fundamental role in the evolution and adaptation of biological organisms and there is evidence to suggest that they can play an equally important role in the design of robotic behavior [5,6,7], their potential role, for purely practical reasons, is often overlooked in favor of control software design [8,9]. The common practice in robotics is to adjust the robot controller to reduce the gap between actual and desired behavior: sensors appear as designer-imposed properties of the robot—expressed in terms of the number, type, and placement of sensors—and not degrees of freedom on which the robot can act to achieve its goal. Although there are approaches in which co-evolution of robot controller and morphology is present [10,11,12,13,14,15], the question of sensor evolution typically entails a particular morphological composition of predetermined sensor building blocks, a specific choice among the combinatorial possibilities of sensors offered by the designer’s sensor library.

Excluding sensor modifications from the design of robots leads to a reduction in the search space of candidate solutions, which ultimately translates into robots that are less fitted to their environment and less robust in maintaining their behavior [16] than biological organisms. Their exclusion also has implications for the degree and evolution [17] of the fault tolerance property of a system. Sensor failures may indeed occur in robots operating in real-time, and controller adaptation mechanisms alone might not be sufficient to cope with such contingencies. In this scenario, the online—during the robot lifetime—evolution of sensors could be a game changer.

So far, since Gordon Pask’s remarkable but unique attempts to evolve real physical sensors in 1958 [18,19,20,21] and the “intrinsic hardware evolution” described by Adrian Thompson [16,17,22,23], little has been carried out in this direction. The primary reason for the paucity of works dealing with the artificial evolution of sensors is due to the impossibility of having real changes in the physical substrate representing the sensory apparatus. The examples of artificial sensor evolution that can be found in the literature are limited—both in number and relevance—and only scratch the surface of the topic of interest.

Olsson et al. [6] proposed an effective but limited sensory adaptation method. Indeed, since it is not possible to expand the sensory capabilities provided by the system designer or change their physical structures, the method tries to optimize its use. More precisely, through Shannon entropy maximization and adaptive binning, the robot continuously adapts its internal transfer function to the structure of the input distribution in order to produce optimal and compressed encoding of sensory information.

In [5], the authors instead presented an offline design method for sensors involving the employment of a genetic algorithm with operators allowed to operate not only on the structure of the control software architecture but also on the positions and ranges of the sensors, all of the same type. Their work suggests an advantage in the combined optimization of controllers and sensors, as robots with the highest fitness were achieved when changes in both were allowed. Moreover, the experiments shed light on another prominent aspect of sensor evolution: economy in the use of resources (precisely sensors in this case). Indeed, even in the absence of a penalty on the number of sensors, the evolutionary algorithm produced robots that used fewer sensors than those available for the task tested.

Similar to the previous work, many other studies in the literature on sensor morphology optimization employ evolutionary algorithms for choosing the range, placement, and type of sensors to be used, such as [10,24], to name a few.

The literature also presents examples of the ontogenetic (online) adaptations of sensory systems [25]. However, again, it translates in practical terms into the choice of a possible instantiation of the parameterized sensory system at the design stage, which involves the number and arrangement of floor sensors in this case.

With regard to soft robotics, an evolutionary algorithm is used in the work [26] to optimize the sensory apparatus of voxel-based soft robots, i.e., to decide on the optimal arrangement of the number, kind, and placement of predefined sensors. In the paper [27], an example of sensor design for conferring proprioceptive capabilities to a soft robot is presented. the work addresses the problem of finding the minimum set of sensors from a large set of producible sensors.

The work presented in [28] investigated how the robot’s morphology, particularly sensor placement, affects the robot’s ability to learn multiple tasks without experiencing catastrophic forgetting.

In [29], a noteworthy work on in situ adaptations of sensor morphology is presented. The study introduces a robotic system that can adapt the end effector of a robot manipulator by extruding thermoplastic adhesive material. Although the work emphasizes the crucial importance of autonomous adaptations of sensor morphology, the process it uses is expensive, specific to the material used and the goal for which the robot was designed. Therefore, it is not easily generalizable to other application scenarios. For a complete discussion of sensory morphology adaptation, the reader is referred to the review paper by [30].

To move from the typical question “what is the optimal arrangement of your sensors?” [31] to designing robots to cope with uncertain environments through mechanisms that allow them to create *their own sensors*, this paper introduces the concept of a “metasensor”. The “metasensor” is a generic architectural component that extends the classical robot model through an additional layer that offers, at the same time, greater or different sensory capabilities to the robot and a computational model suited to accommodate a process of sensor evolution reminiscent of that taking place in living organisms.

The main objective of this work is to introduce the metasensor architectural component and begin to demonstrate its potential in conferring robotic agents and its ability to adapt more effectively to their assigned tasks—even in the presence of dynamic and unknown environments—through the evolution of ad hoc interpretations of its input signals. The novelty of this work lies precisely in the metasensor’s ability to freely shape perceptual processes according to the robot’s needs and contingencies, without a priori constraints imposed by the designer. The flexibility and plasticity offered by the architectural layer that implements the metasensor allow an evolution of the sensory subsystem that represents a novelty compared to traditional robotic sensor design methodologies found in the literature.

This article is organized as follows: Section 2 is devoted to the presentation of the metasensor model and the problems it aims to overcome. Section 3 describes the relationship between the metasensor and the research fields of cybernetics and biosemiotics. Section 4 presents a proof of concept of the metasensor model in a simple but paradigmatic robotic scenario, demonstrating the potential and applicability of the proposed concept. In particular, two different implementations of the metasensor are proposed: one hand-coded and another based on a neural network, whose weights are evolved through a genetic algorithm, producing a first example of sensor evolution in a robotic system. Section 5 discusses the results obtained and contextualizes them in the more general context of possible real-world application scenarios of the metasensor, highlighting limitations of the current implementation and possible improvements for future work. In addition, some visionary applications involving the use of the metasensor are discussed. Finally, Section 6 concludes this manuscript.

## 2. Metasensor Model: Control by Interpretation

As can be appreciated from the examples presented in the Introduction, artificial sensor evolution is not an “all or nothing” property, but, rather, a characteristic of a system for which a degree of attainment can be defined, which depends on the designer’s choices and in turn on the possibilities offered by the robot model used. In fact, the evolution of sensors in artificial systems is a process that must be enabled, and fostered, explicitly by the designer, not a “for free” emerging property provided by natural selection, development, and learning processes, as is the case of the biological counterpart. It then becomes necessary to design an architectural component that can accommodate the artificial evolution of sensors, possibly requiring minimal human intervention and being generic enough to be suitable for various application scenarios.

**Figure 1:**
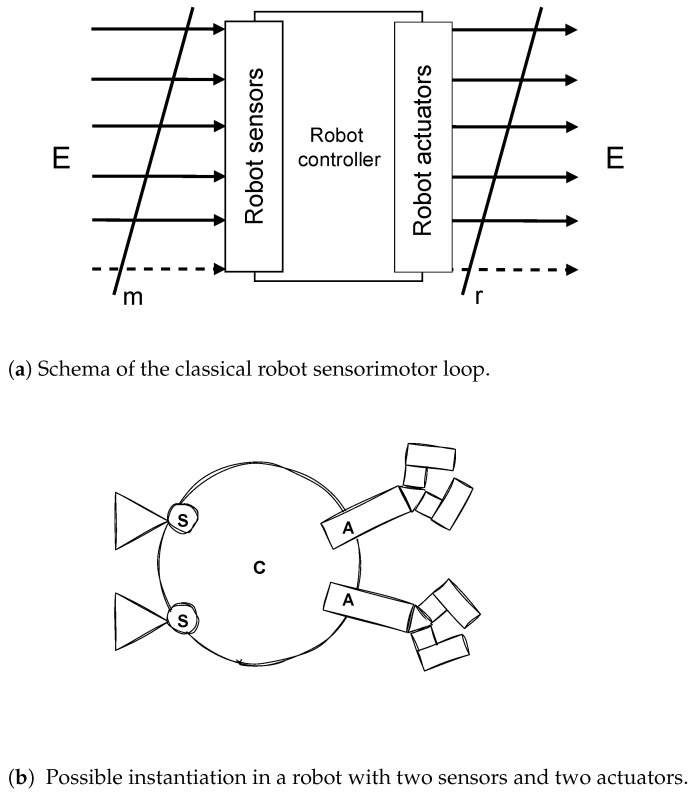
Classical robot sensorimotor loop. (**a**) Abstract representation of the components of the sensorimotor loop of a robotic agent. (**b**) Schematic representation of a robot with a sensorimotor loop composed of two sensors (S), two actuators (A), and a control software (C). The control software processes the input signals from the sensors and produces the output signals that drive the actuators. The letter E stands for “environment”.

**Figure 2:**
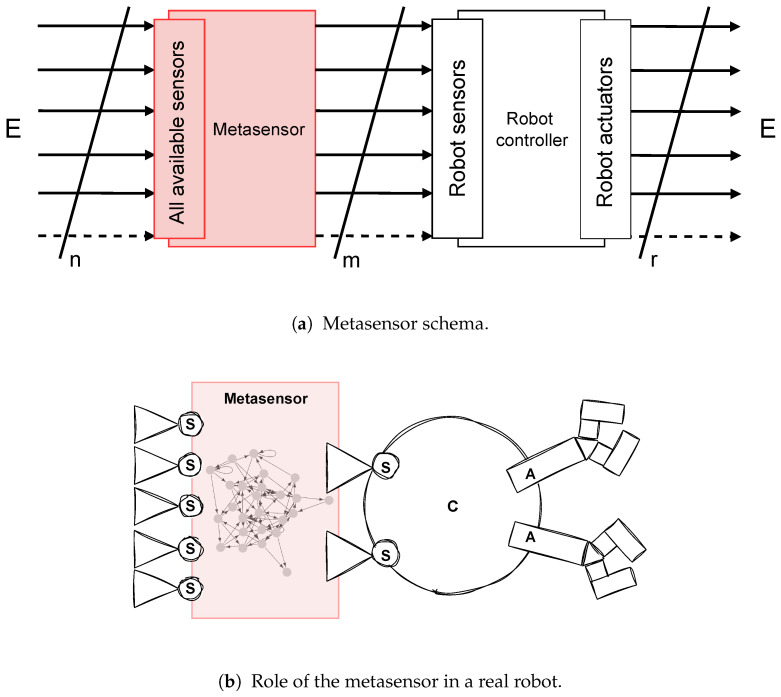
The role of the metasensor in the robot sensorimotor loop. (**a**) Diagram representing the role of the *metasensor* in the robot sensorimotor loop: in short, it enhances the sensory capabilities of the robot by increasing the number and type of sensors and offering memory-based processing. (**b**) Possible instantiation of the metasensor concept with the robot proposed in Figure 1b; in the rectangle, a possible reification of the metasensor processing unit in the form of a recurrent network is illustrated. The letter E stands for “environment”.

For these reasons, the novel concept of a **“metasensor”** is introduced here for the first time. Figure 1 depicts the classical robot sensorimotor loop, while Figure 2 reports its architectural change imposed by the introduction of the metasensor.

From the schema reported in Figure 2a, it can be noted that the metasensor is composed of two parts: (*i*) a hardware interface responsible for extending or changing the robot **sensing** through the introduction of a series of physical sensors capable of capturing signals coming from different phenomena present in the environment; (*ii*) a software computational module responsible for the **perception** phase which allows higher order processing and therefore the integration, interpretation, and organization of the captured sensory information.

Referring to Figure 2a, the hardware component extends the vector of robot input signals $I={\left[\begin{array}{c} I_{1},I_{2},\cdots ,I_{m} \end{array}\right]}^{T}$ from *m* to *n* dimensions. It is also possible that not all of the *m* sensors of the robot are controlled by the metasensor. In this case, some sensors will continue to capture signals from the surrounding environment. In principle, we could state that the more generic the sensors are (i.e., with input values in a wide range and possibly without any kind of signal preprocessing), the more freely the mechanism of sensor evolution that takes place in the metasensor can explore new correlations between signals and actions. Thus, it can create new signs and, in turn, new meanings useful for successfully attaining any given task. Ideally, a suitable generic sensor for this type of application would be a broadband sensor capable of capturing electromagnetic waves over a wide range of frequencies, such as the one reported in [32], a photonic electric field sensor capable of capturing a frequency range from 10 MHz to 26.5 GHz. Clearly, there must be compatibility between the metasensor’s output interface and the robot’s input interface: in other words, the metasensor must respect the physical constraints imposed by the robot by producing output signals that can be interpreted by its sensory modalities. For example, a metasensor connected to a robot equipped only with light sensors will have to emit light signals in order to control it.

On the software side, the metasensor autonomously performs a **model-free** search in the space of “perceptual processes” with the goal of finding the optimal interpretation of its input signals to feed to the robot to make it perform the given task. To this end, although , in principle, there are no constraints on the model that can reify the metasensor at a practical level, subsymbolic models are an ideal representation of it, as they operate at a lower level of abstraction than symbolic models and can leave full freedom to evolution to achieve the best configuration for the particular scenario at hand. The metasensor dynamically changes—through evolution/adaptation/self-organizing mechanisms—the way it processes the input signals just captured by the hardware interface. The outputs computed will in turn become the robot’s inputs and act on it by perturbing its dynamics. The meaning (semantics) attributed to the input signal by the metasensor will be contingent on the specific environment, task, robot model, and the specific affordances [33,34,35] that become available over time. The type of control exerted by the metasensor on the robot can be referred to as “control-by-interpretation”: the metasensor tries to find the appropriate interpretation of the input signals able to exploit the bouquet of dynamics that the robot can exhibit to steer it toward the desired behavior. It is precisely the ability to perform a model-free search in the space of perceptual processes that endows the entire robot-metasensor system with a *semantic adaptivity* and *the ability to evolve its own sensors* to cope with the contingent situation. The metasensor acts from a semiotics perspective as a meaning-making subsystem. Indeed, sensor evolution allows the robot to successfully operate in unpredictable and dynamic environments: the metasensor can change—as a function of a potentially changing over time objective function—to deal with the contingencies as they arise. Its ability to adapt to changing conditions enables the robotic system to implicitly adapt to environmental peculiarities and idiosyncrasies. This also encompasses the ability to adapt to absorb transient and systematic errors and delays affecting input readings and signal transmission, which may occur as a result of changed environmental conditions and malfunctions or physical damage occurring to the robot itself [36].

Unlike the classical robotic scheme shown in Figure 1a, the signals that perturb the robot are now influenced not only by the robot’s environment and actions but also by the internal dynamics (the process of meaning creation) of the metasensor. This can be formalized as follows:

$$O_{M}\left(t+1\right)\equiv I_{R}\left(t+1\right)=f\left(E\left(t\right),U_{M}\left(t\right),A_{R}\left(t\right),t\right)$$

where $O_{M}=\left(o_{1},o_{2},\cdots ,o_{m}\right)$ and $U_{M}$ refer to the output vector and the state vector of the metasensor, respectively, while E represents the state of the environment. Additionally, $I_{R}$ and $A_{R}=\left(a_{1},a_{2},\cdots ,a_{r}\right)$ are the inputs controlled by the metasensor and the action vector of the robot. Since the metasensor can retain the memory of its past, the state vector $U_{M}$ can depend on its previous state, going beyond the concept of a dynamical multiplexer:

$$U_{M}\left(t+1\right)=g\left(E\left(t\right),U_{M}\left(t\right),U_{M}\left(t-1\right),\ldots ,U_{M}\left(0\right),A_{R}\left(t\right),t\right)$$

The dependence of *f* and *g* functions on time accurately reflects the possibility of a contingent evolution of the metasensor. In summary, the dynamics of the metasensor, together with the environment and the robot’s own past actions, determine the sensory inputs of the controlled robot. This will in turn affect the robot’s internal state and its subsequent actions, thereby closing the causal feedback structure—between environment, metasensor, and robot—that drives the robot’s observable emergent behavior. From the perspective of information theory, the metasensor acts on signal interpretation to reduce uncertainty about the input signal itself.

The metasensor can also be framed within the framework of the free energy principle [37,38]. According to this perspective, the metasensor acts to minimize the variational free energy of the system by assuming a dual role. On the one hand, it can play the role of the likelihood model: the controlled robot intensively encodes the a priori probability distribution since it is a dynamical system, and as such its basins of attraction—their relative sizes in particular—confer the system the ability to classify a given input signal [39]. In this view, the evolution of the metasensor changes the perception of the whole system in that it allows a perturbation (an input signal) to “flow into” one basin of attraction, rather than another, depending on its goals. At the same time, on the other hand—being a dynamical system in its own right—the basins of attractions of the metasensor extend/modify those of the robot controller, thus changing the a priori distribution of the system as a whole.

The sensor evolution mediated by the metasensor is a complementary approach to robot control software design. However, it offers the advantage of being able to operate in application scenarios where changing the control software is problematic for various reasons. The advantages become apparent when it is impossible to change the controller of an already operating robot in the real world either because it is deemed too expensive or because the controller’s internal model is inaccessible. In those cases, the metasensor represents a viable control mechanism for modifying the robot’s behavior while leaving its controls software unchanged, even and especially in online scenarios, i.e., taking place during the robot’s lifetime [40,41].

Arguably, a metasensor equipped with a simple sensor, such as a temperature sensor, to control a Kilobot robot [42] is the most basic but paradigmatic example of a possible robotic system following the proposed approach. In its simplest incarnation, the metasensor controls the Kilobot by sending it signals—in the form of light signals—of an intensity proportional to the temperature signals detected by the metasensor, i.e., without any further signal processing. Despite its simplicity, this case study highlights an important aspect of the proposed architectural component, namely the possibility of modifying the robot’s sensory modalities. the resulting system, metasensor and Kilobot, can now address several application scenarios otherwise precluded to the robot alone. Examples of those include the following: (*i*) fire rescue operations to detect areas of intense heat, helping firefighters to locate and extinguish flames more efficiently; (*ii*) for the agricultural environment, monitoring the microclimate around plants and vegetables and thus maintaining ideal growing conditions by adjusting soil temperature with swarm formations of Kilobots to maintain shading in case of excessive temperature; and (*iii*) for research and exploration in hostile environments, such as space, enabling temperature mapping to detect thermal variations detrimental to the main rovers and detection of the presence of ice.

Instead, to uncover the true application potential of the metasensor, i.e., the possibility of sensory evolution, we must resort to the use of broadband sensors, such as optical spectrometers, broadband acoustic sensors (e.g., microphones), and broadband electromagnetic sensors. In this case, the possible application scenarios of a system consisting of a metasensor with such broadband sensors and a robot are limited only by the actual range of values captured by the sensors, the robot’s actuating capabilities, and the capacity of the transmission channel—i.e., metasensor and robot controller—on which adaptation mechanisms such as those presented in the following works [40,41,43], as already mentioned, can act to maximize the mutual information between input and output.

## 3. Between Cybernetics and (Bio)Semiotics

From the argumentation provided in the previous sections, it is evident how the action of the metasensor is aimed at controlling a robotic agent by allowing it to express different behavior from that for which it was designed, and thus how this component acts as a cybernetic component. Indeed, it plays a fundamental role in determining “what the robot does”. In this sense, we can frame the metasensor as a generalization of Arkin’s Perceptual Schema [44], Selfridge’s Pandemonium architecture [45] and, in general, the sensory input layer of many other control schemas that fall under the cybernetic discipline.

However, if we ask “what is” a metasensor, it is inevitable to state that it is the component in which the phenomena of signification reside and occur. Thus, its semiotic role in the robot-environment relationship becomes evident. Cariani, in his studies [18,46,47,48], had already stressed the importance of the creation of meaning and appropriate *relevance criteria* in artificial agents and how they foster the creation of autonomous agents, in the true sense of the word. In part, while not defining a model, Cariani himself had historically anticipated the role and importance of the metasensor, speaking of “open-ended neural networks” for creating “semantically adaptive artificial devices”.

It is, however, with the conceptual decoupling of the metasensor and robot controller and with the following recently published work [40,41,43] that the foundations have been laid for achieving the evolution of sensors in artificial contexts. These works indeed paved the way for the realization of the metasensor layer from a practical point of view, as they present the possibility of implementing a non-destructive reversible adaptation process acting on a raw substrate, e.g., Boolean networks and nanowire networks [49], in robotic contexts, in order to achieve different behaviors. By acting only on the sensor–node and node–actuator couplings without altering the structure of these models, the authors showed that robots can show interesting behaviors. Thus, the same unchanged network, and especially the bouquet of dynamics it can express, can be reused to make the robot controlled by it perform other novel behaviors.

The same process is at the basis of metasensor functioning, but what is gradually crafted by the adaptation process is not the control software but the interpretation of the input signals that will later feed into the robot’s control software. The evolution in the case of a metasensor works in the space of symbols received by the input sources and on the already processed chunk of symbols, generating, depending on the specific circumstances, information processing processes useful for obtaining the desired behavior. This process can lead to the creation of new internal symbols and new interpretations of old symbols, thus extending the syntactic and semantic vocabulary of the metasensor-robot system [34]. In short, we can say that the evolution of sensors in the metasensor assumes the form of the search in the space of *epistemic functions* [50].

## 4. Proof of Concept

To begin to unveil the potential of the metasensor in robotics, a paradigmatic proof of concept is presented in this section. In the full spirit of the metasensor, the proof of concept application presents a robotic system—initially designed to perform a given task—whose behavior is to be modified by the presence of the metasensor. Thus, we consider a Braitenberg-like agent as the robot to be controlled, which performs a task of avoiding sources of light (i.e., the “fear” behavior) [51]. At this point, a metasensor is added to the robot, which will be responsible for imparting a different behavior to the robot than the original one. In fact, as stressed in the previous sections, the metasensor can extend and evolve the robot’s sensory system and, among other things, enable it to perform a task other than the one for which it was designed. The task under consideration in this section then becomes the following: changing the robot’s behavior from a light avoidance task to an area avoidance task.

NetLogo 6.4.0 (Northwestern University, Evanston, IL, USA) [52] is used to implement the proof of concept. Although NetLogo is not a primary choice for robotic modeling and simulation, it was chosen because of its simplicity and, above all, because its abstraction allows us to faithfully reflect the conceptual separation between the metasensor and the controlled robot. In effect, the metasensor and the robot can be implemented in NetLogo as two different breeds of agents, allowing them to be two distinct components, thus replicating the formal and physical decoupling of the two systems. This allows us to better demonstrate the capabilities and potential of the metasensor, as previously theorized (The same example can be translated into a real robotics application—e.g., using the ARGoS simulator [53] and a foot-bot robot model [54]—but, in this case, the metasensor concept must be implemented as an additional perceptual scheme of the robot, reducing the effective degree of decoupling between the two subsystems).

**Figure 3:**
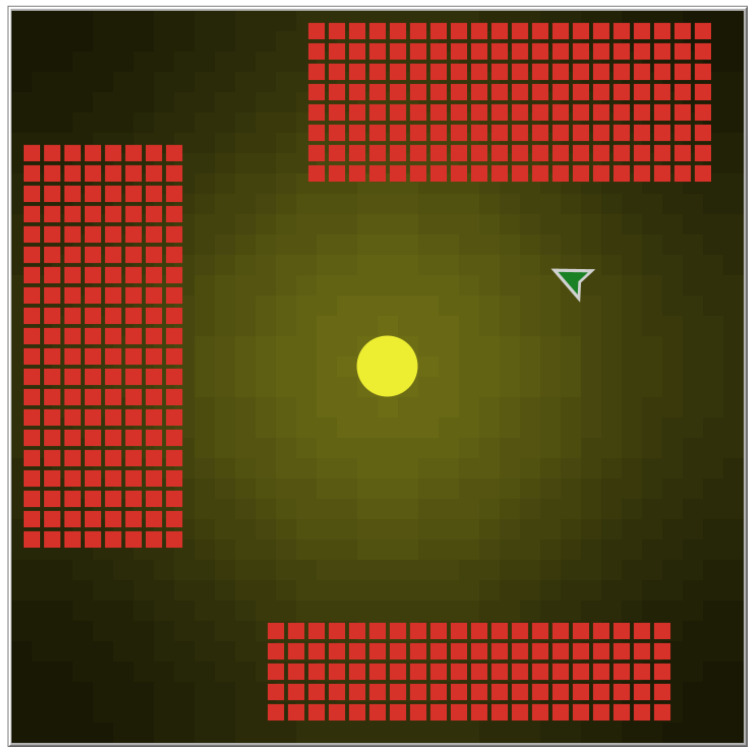
The arena used for the examples features a light source (yellow circle) in the center and three red-colored areas covering approximately one-third of the entire arena. The robotic agent is represented by a gray arrow while the metasensor is represented by a green arrow and is located above the robot, just as it would be in its actual physical implementation. The metasensor, once added, will form a unique overall system with the robot and will therefore be an integral part of it and move with it.

As said before, the robot taken into account as a starting point for the proof of concept is a Braitenberg vehicle that avoids light sources. The arena will consequently present a light source (a yellow circle) in the center, whose light spreads out to create a light gradient, maximum at the center and gradually decreasing as you move away from it. The arena is a torus, so the robot can wander without encountering obstacles. Since the metasensor has to change the robot’s behavior from light avoidance to area avoidance, three red areas are added to the arena. The arena used in the experiments is shown in Figure 3.

A light-avoiding Braitenberg vehicle has the following connections between sensors and actuators:

- The left sensor is connected to the left wheel;
- The right sensor is connected to the right wheel;
- The speed of the wheels is proportional to the intensity of the light detected by the sensors.

**Figure 4:**
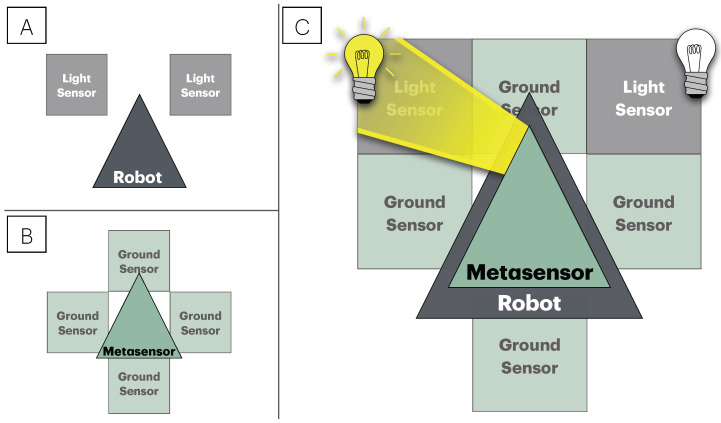
Robot and metasensor models implemented in NetLogo with their sensory modalities. (**A**) Graphical schematic representation of the robot model with its sensors. The robot is schematized by a gray triangle, and its sensory equipment consists of two light sensors positioned in front of it, one on the left and one on the right. (**B**) Graphical representation of the metasensor model and its sensors. The metasensor is represented as a green triangle. The metasensor’s sensor apparatus consists of four ground sensors located to the north, west, east, and south of the metasensor, respectively. (**C**) Representation of the whole system composed of the robot and metasensor subsystems along with the interaction between the two. The metasensor is located above the robot and interacts with it by acting on its light sensors, i.e., the metasensor can modify the light intensity perceived by the robot according to its sensory input and internal state, thus changing the robot’s behavior. The figure also depicts the type of interaction that takes place between the metasensor and the robot: the modulation of light intensity and in particular the differential light between the robot’s left and right sensors perceived by the robot and produced by the metasensor in response to the presence of the red areas in the arena will cause a change in the robot’s direction of movement and thus a modification of its original light-fearing behavior. As an example, in the figure, the light beam is emitted by the metasensor and stimulates the robot’s left light sensor.

Therefore, we model our robot as a simple NetLogo agent mimicking Braitenberg’s robot. Indeed, although there are no real wheels in our NetLogo agent, we simulate their presence by moving against the light gradient. Thus, if the light sensor on the left perceives more light than the one on the right, the agent will turn to the right, and vice versa. The two sensors are practically implemented by the two patches in front of the agent, to its left and right, respectively. The model of the robot is represented by a gray triangle shape; Figure 4A presents the robot model with its sensors.

Since the metasensor is a generic component and can be applied to any robot model, we must define its output interface to be compatible with the robot’s input interface. In this case, the metasensor will act on the robot by changing the light intensity perceived by the robot’s sensors, acting on the differential level of light perceived by the left and right sensors. Furthermore, to change the robot’s behavior from light avoidance to area avoidance, the metasensor will have to detect the presence of the red areas in the arena. So, the metasensor is equipped with four ground sensors located to the north, west, east, and south of the metasensor, respectively. As a result, the metasensor will modulate the light intensity perceived by the robot according to the input signals received from the ground sensors.

The metasensor model is represented by a green triangle (see Figure 4B). Figure 4C shows the whole system composed of the robot and the metasensor subsystems, along with their sensors and the kind of interaction between the two. By way of example, the light beam depicted represents the light emitted by the metasensor and stimulation of the robot’s left light sensor.

In the following, two different implementations of the metasensor are presented. The first example presents a human-designed metasensor; in the second example, an automatic design process is performed to obtain a functioning metasensor. The first one, albeit simple and based on a priori knowledge of the robot’s original behavior, aims to demonstrate the practical feasibility of the proposed formal metasensor concept. The second represents the first example of a true sensor evolution process in a robotic system and puts the metasensor in a scenario close to its real-life use cases while emphasizing its generality, i.e., the fact that it is not necessary to know in advance the behavior of the robot to which it is connected.

### 4.1. Example 1: Human-Designed Metasensor

As a first kind of metasensor implementation, a human hand-coded version is proposed. The one we present represents the simplest possible version of metasensor implementation, in that it will exploit the knowledge of the original robot’s behavior to change it. At the same time, it is necessary to demonstrate the practical feasibility of the proposed metasensor concept.

The original robot, based on the light intensity perceived by its left and right sensors, moves towards the less intense light source. In other words, it moves against the gradient of light intensity. Thus, the set of rules devised to incarnate metasensor logic exploits this knowledge by increasing the light on the position of the robot’s sensors if the robot is in or is approaching a red area, thus causing the robot to move away from red areas. In particular, the metasensor will increase the intensity of the light perceived by the sensor on the right (left) side of the robot if the ground sensor of the metasensor on the east (west) side is activated. Instead, we decided to break the symmetry of possible choices when both ground sensors on the east and west are activated, increasing only the light sensor on the right. The ground sensor located to the north acts by increasing the intensity of light perceived by the robot’s sensors, if activated. In addition, a random component is added to the light intensity produced by the metasensor if any ground sensor is activated. The full set of rules is presented in the pseudocode of Algorithm 1.
**Algorithm 1** Simple hand-coded metasensor area avoidance.  1:**procedure**               MetasensorAreaAvoidanceHandCoded(robot_sensor_dx, robot_sensor_sx, ground_north, ground_east, ground_south, ground_west)  2:      delta ←0.1  3:      any_sensor_activated ← **false**  4:      increment ←(1+0.5×ground_north)×delta  5:      **if** ground_east = 1 **and** ground_west = 0 **then**  6:            set robot_sensor_dx ← robot_sensor_dx + increment  7:            set any_sensor_activated ← **true**  8:      **else**  9:            **if** ground_east = 0 **and** ground_west = 1 **then**10:                set robot_sensor_sx ← robot_sensor_sx + increment11:                set any_sensor_activated ← **true**12:            **else**13:                **if** ground_east = 1 **and** ground_west = 1 **then**14:                      set robot_sensor_dx ← robot_sensor_dx + increment15:                      set any_sensor_activated ← **true**16:                **end if**17:            **end if**18:      **end if**19:      **if** any_sensor_activated = **true then**20:            set robot_sensor_dx ← robot_sensor_dx + RandomFloat(0.1) − 0.0521:            set robot_sensor_sx ← robot_sensor_sx + RandomFloat(0.1) − 0.0522:      **end if**23:**end procedure**

It is worth noting that this simple set of rules succeeds in altering the robot’s behavior from light avoidance to area avoidance. We quantitatively evaluate the performance of the metasensor by measuring the number of passes of the robot-metasensor system over the prohibited areas, using the following formula:

(1) $$E_{\mathrm{test}}=\sum_{i=0}^{T}\lambda \left(i\right)$$

where

$$\lambda \left(i\right)=\left\{\begin{array}{ll} 1 & \mathrm{if}\;\mathrm{the}\;\mathrm{robot}\;\mathrm{is}\;\mathrm{on}\;\mathrm{forbidden}\;\mathrm{areas}\;(i.e.,\;\mathrm{over}\;\mathrm{red}\;\mathrm{patches})\\ 0 & \mathrm{otherwise} \end{array}\right.$$

Obviously, the lower the value of $E_{\mathrm{test}}$, the better the performance of the metasensor. So, by running the hand-coded version of the metasensor for 10 different simulations, we obtained an average value of $E_{\mathrm{test}}=77.9\pm 31.5$.

A video demonstration of the behavior produced by the hand-coded version of the metasensor is available at the following link: https://github.com/mbraccini/Metasensor_Sensor_Evolution_in_Robotics, accessed on 10 January 2025.

### 4.2. Example 2: Automatic Design of Metasensor

**Figure 5:**
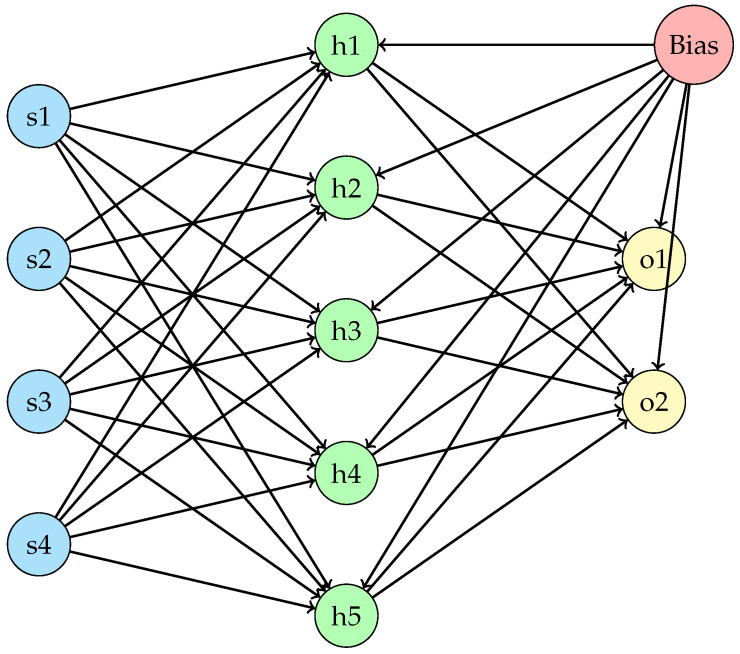
The neural network architecture used to implement the metasensor in the case of automatic design. The network is feed-forward and consists of three layers: an input layer with four nodes $\left\{s_{1},s_{2},\cdots ,s_{4}\right\}$, a hidden layer with five nodes (with sigmoid activation function) $\left\{h_{1},h_{2},\cdots ,h_{5}\right\}$, a bias node, and an output layer with two nodes (with linear activation function) $\left\{o_{1},o_{2}\right\}$.

In the second example, to actually evolve the sensory apparatus, the metasensor is automatically designed by an evolutionary algorithm, a genetic algorithm (GA). (As stressed in the introduction, this represents only one specific form of sensor evolution process; other possibilities include adaptation and learning, offline or online). Thus, in order to confer plasticity on the substrate that implements the metasensor and thus effectively evolve the sensors useful for producing the area avoidance task, a neural network is introduced as the processing unit. The neural network used for this proof of concept is a simple feed-forward three-layer network with four input nodes (for the four ground sensors), five hidden nodes, two output nodes (for the production of the two light signals then read by the robot) and a bias node. The activation function used is the sigmoid function for the hidden layer, while a linear function is used for the output layer. The architecture of the neural network is shown in Figure 5. The use of evolutionary computation to optimize a neural network—in this case used as a substrate for the metasensor—is a common approach in evolutionary robotics [55]. Evolutionary robotics employs population-based algorithms inspired by natural selection to optimize the control policies and morphologies of autonomous robots. A fitness function is used to guide the selection process. The particular neural network architectural model employed in this study draws inspiration from the seminal contributions to robotics by Nolfi et al. [56].

The neural network is then fed with the four input signals from the sensors on the ground and produces two output signals that will be used to modulate the light intensities then perceived by the robot’s sensors.

In this specific case, the metasensor must find a coherent and effective set of neural network weights capable of orchestrating metasensor-robot behavior to perform the desired area avoidance behavior. A genetic algorithm is put in place to perform this task, namely the search in the space of neural network weights. The parameters used for the genetic algorithm are summarized in Table 1. The particular choice of parameters for the GA is grounded in well-established principles and widely recognized best practices in the field [57]. The chosen scheme can be described as a balanced exploration–exploitation strategy. In particular, with a crossover rate of 0.6 and a mutation rate of 0.4, GA ensures good recombination of promising solutions while maintaining genetic diversity. the same applies to the number of generations and the population size, both set to 100 to achieve good initial diversity and a high probability of convergence to high-quality solutions, while keeping the computational time relatively low. Furthermore, tournament selection ensures good selective pressure. However, as the aim of this work is not to find the best metasensor configuration, but to demonstrate the feasibility of its automatic design, no further comparisons were made. The decision not to conduct further experiments is also supported by the excellent performance of the solutions found, described in detail in the Results Section 4.3. The genetic algorithm was implemented in Python using the DEAP library [58], particularly using the eaMuPlusLambda function for the evolution process. The value of μ equal to 25% of the population size is chosen to have a high selective pressure, while λ equal to 100% generates a number of offspring equal to the original population size and thus, in concert with the probability of crossover and mutation used, maintains a high genetic diversity during the search process.

The fitness function used to evaluate the performance of the robot-metasensor system and to guide the evolutionary algorithm is the following:

(2) $$F_{o}=\frac{1}{T}\sum_{n=0}^{T}\left(\sqrt{v(n)}\cdot \left(\frac{1}{1+\frac{H(n)}{n+1}}\right)\cdot \gamma \left(n\right)\right)$$

where

- *n* is the actual simulation step.
- T is the total number of simulation steps.
- γ(n)∈{0,1} return 1 if the robot is on prohibited areas (i.e., if at least one ground sensor is 1), 0 otherwise.
- v(n) is the speed of the robot at the n-th step, with values in [0,1].

In our experiments, *T* is set to 1000 steps.

For practical reasons, we used information derived from the NetLogo reference system to estimate the movements of the metasensor-robot system; in particular, we estimated the steering angle and speed of the entire system. In a real robot, this information can be retrieved by equipping the metasensor with an additional positioning sensor, such as a GPS. So, H(n) is the sum of the minimal angular differences until the n-th step and it is defined in the following way:

$$H\left(n\right)=\sum_{i=1}^{n}\left|\Delta \theta_{i}\right|=\sum_{i=1}^{n}\left|\left(\theta_{i}-\theta_{i-1}+180\right)\;\mathrm{mod}\;360-180\right|$$

$\Delta \theta_{i}$ reduces the turns that exceed $\pm 180^{\circ }$ to the range [−180,180].

The fitness function is designed to reward the robot for moving fast and in a straight line while avoiding the prohibited areas, and to penalize it if it moves slowly and turns often.

### 4.3. Results

In this section, the results of the automatic design of the metasensor are presented. The genetic algorithm is run for 100 generations with a population size of 100 individuals. The total number of simulation steps is set to 1000, so the fitness function is evaluated 1000 times for each individual. A total of 30 independent runs are performed to start assessing the robustness of the results. The genetic algorithm parameters are shown in Table 1.

**Figure 6:**
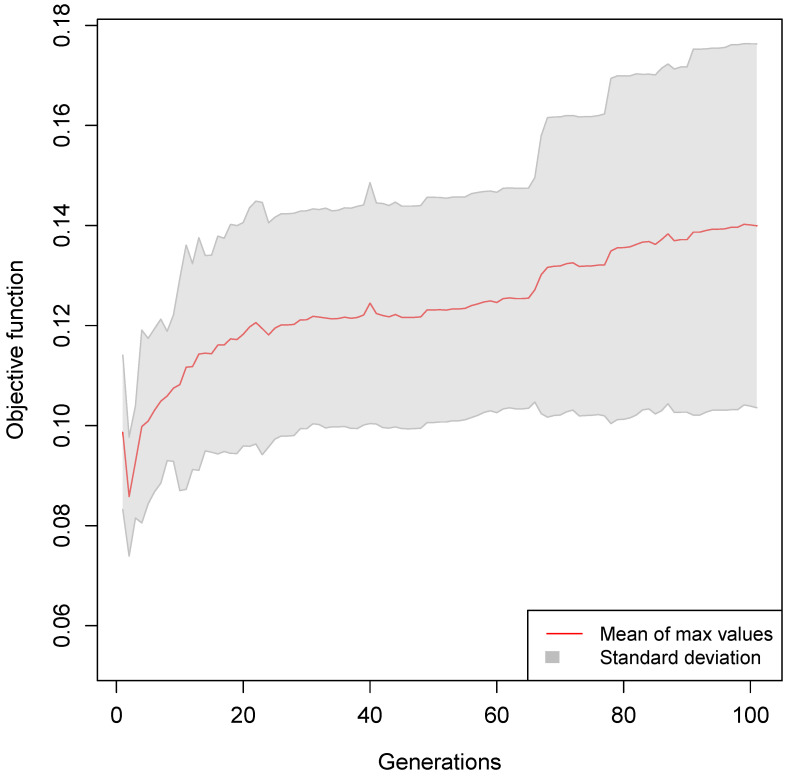
The trend of the mean ± standard deviation (gray area) of the maximum fitness values for each epoch over the 100 generations in the 30 independent runs of the genetic algorithm.

The mean of the maximum fitness values found by the genetic algorithm over the generations is shown in Figure 6. The trend of the maximum fitness values is increasing, showing that the genetic algorithm is able to find better solutions over time.

**Figure 7:**
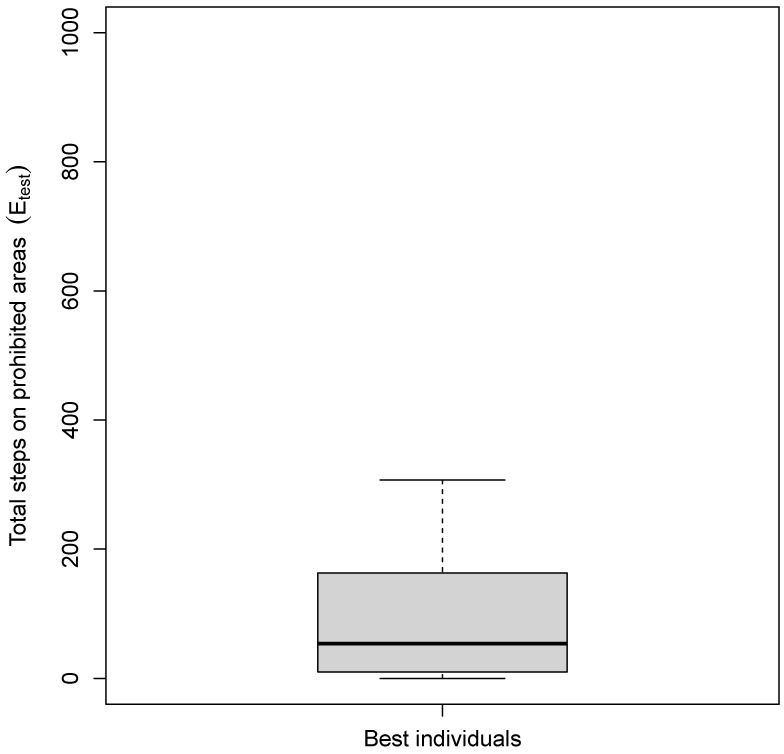
Boxplot showing the total steps in the prohibited areas (i.e., $E_{\mathrm{test}}$) of the 30 solutions found by the genetic algorithm.

The 30 solutions found are then tested to see if they actually learn to perform the area avoidance task by evaluating their performance with Equation (1).The statistics collected for this last analysis are summarized by a boxplot, showing the total steps in the forbidden areas for the 30 solutions found by the genetic algorithm (see Figure 7). The results show that the solutions found successfully avoid the prohibited red areas, as they spend 53.5 steps (out of a maximum of 1000), which is the median value in these areas in total during the test simulations. This is a very promising result, considering that the red areas occupy one-third of the total arena and are located in places where the robot initially often passes, due to the robot’s original behavior. This demonstrates the effectiveness of the automatic design process and especially the potential of the metasensor to evolve its perception processes—and thus the sensory system—to ultimately modify the robot’s behavior.

A video of the behavior produced by one of the best metasensor solutions produced by the genetic algorithm can be found at the following link: https://github.com/mbraccini/Metasensor_Sensor_Evolution_in_Robotics, accessed on 10 January 2025.

## 5. Discussion

The proof of concept demonstrates the feasibility of the proposed metasensor concept and emphasizes two of its fundamental characteristics: (*i*) the ability to evolve a sensory apparatus using an artificial medium (in this case, a neural network) and to (*ii*) modify the behavior of a robotic agent.Although the current implementation may not fully reflect real-world scenarios, the results obtained in the case of automatic metasensor design are very promising and open up new perspectives for the application of the metasensor in real robots, even in online adaptation scenarios.

Future work will precisely be devoted to the development of application scenarios to test and appreciate its potential in real robots and in online adaptation scenarios. Indeed, as previously discussed, the properties of the metasensor meet the requirements, needs, and constraints imposed by the online adaptation of robots. Thus, case studies can be envisioned in which, during the robot’s lifetime, a robot is assigned a task for which it lacks the sensory modalities necessary for its successful accomplishment. So, the metasensor has to adapt its perception—through a reorganization of the set of information processing dynamics—to provide appropriate processing of the new information to accomplish the new task, while syntactically respecting the constraints expressed by the robot’s sensory interface. To cope with the complexities of online adaptation, the substrate that implements the metasensor may require memory and other properties that characterize biological organisms, such as robustness and flexibility. Therefore, systems that operate in a critical dynamical regime, such as critical Boolean networks [43], could potentially be a starting point for metasensor designers. Alternatively, nanowire networks [40] could be a further design solution if the goal is to build cost-effective, miniaturized robotic systems. In light of the potential outlined, it is reasonable to conclude that the metasensor can therefore play a decisive role in the development and advancement of fully automatic online design techniques [59].

The features of the metasensor implicitly address the issues of optimization and reuse of the available resources, minimizing costs since it avoids a complete redesign of robotic agents. It is therefore not difficult to envision a future where discarded and obsolete robots of different morphologies and functionalities can be brought “back to life” through the use of metasensors.

Visionary applications of the metasensor involve robotic applications in hostile environments, where groups of simple (micro)robots must complete a given mission. Here, individual (or groups of) robots may (*i*) need to change the subtask they face over time and (*ii*) be subject to possible sources of information not foreseen in advance by the designer, or a combination of the two. In any case, the metasensor provides the robot with the ability to flexibly adapt its behavior on the fly. In this application scenario, the metasensor can facilitate the emergence of heterogeneity in groups of robots due to sensor specialization, thus enabling the emergence of different sets of behaviors in the group, each specializing in a specific subtask. Also, the sensor specialization provided by the metasensor can work in synergy with evolutionary algorithms to accelerate the development and deployment of (possible new declinations of) real-world evolutionary robotics [60,61,62].

Lastly, since it can be implemented on physical hardware, the metasensor offers the possibility to investigate whether it is possible to overcome in robotic applications the concept of combinatorial novelty, i.e., the same emergent phenomenon that evolution triggers in Thompson’s evolvable hardware.

## 6. Conclusions

This paper presents a novel robotic architectural component, the metasensor, capable of reproducing a process of sensor evolution in a robotic system, reminiscent of what occurs in living organisms. Through the evolution of perceptual processes, the metasensor can modify the robot’s original behavior and make it semantically adaptive, thus extending the robot’s capabilities and adaptability. Thanks to the metasensor, the robot can adapt to new tasks and unexpected situations without the need to completely redesign the robot’s hardware and software, thus providing a cost-effective and efficient solution to robotic challenges, even in dynamic and unpredictable environments.

The promising experimental results obtained with proof of concepts validate the feasibility of the metasensor and highlight its potential for real-world applications. Although further research and experiments in more complex arenas and tasks are needed to fully test its limits and potential, as well as to assess its reliability, safety, and robustness, the metasensor stands as a promising solution to tackle not only current but also future challenges in robotics.

## Figures and Tables

**Table 1:** Genetic algorithm parameters.

Parameter	Value
Crossover Rate	0.6
Mutation Rate	0.4
Number of Generations	100
Population Size	100
Selection Strategy	Tournament selection (size 3)
Crossover Type	Two-point crossover
Mutation Type	Gaussian mutation (μ = 0, σ = 1)
μ	25% of population size
λ	100% of population size

## Data Availability

The original results and software presented in this work are available in Zenodo at https://doi.org/10.5281/zenodo.14412247, accessed on 10 January 2025.
